# Supplementary material for: Tead1a Initiates Transcriptional Priming Through the TEAD1a/YAP‐Notch1‐Spi1/Cebpα Axis to Promote Neutrophil Fate
Source: Adv Sci (Weinh). 2025 Aug 28;12(41):e05441. doi: 10.1002/advs.202505441 (PMC12591127; doi:10.1002/advs.202505441)
Supplement: Supplementary file 4 — Supplementary Dataset [file ADVS-12-e05441-s004.zip › 3-8.zip!/3-8/0904/0904/05.annot/Neutrophils/featureplot.pdf]

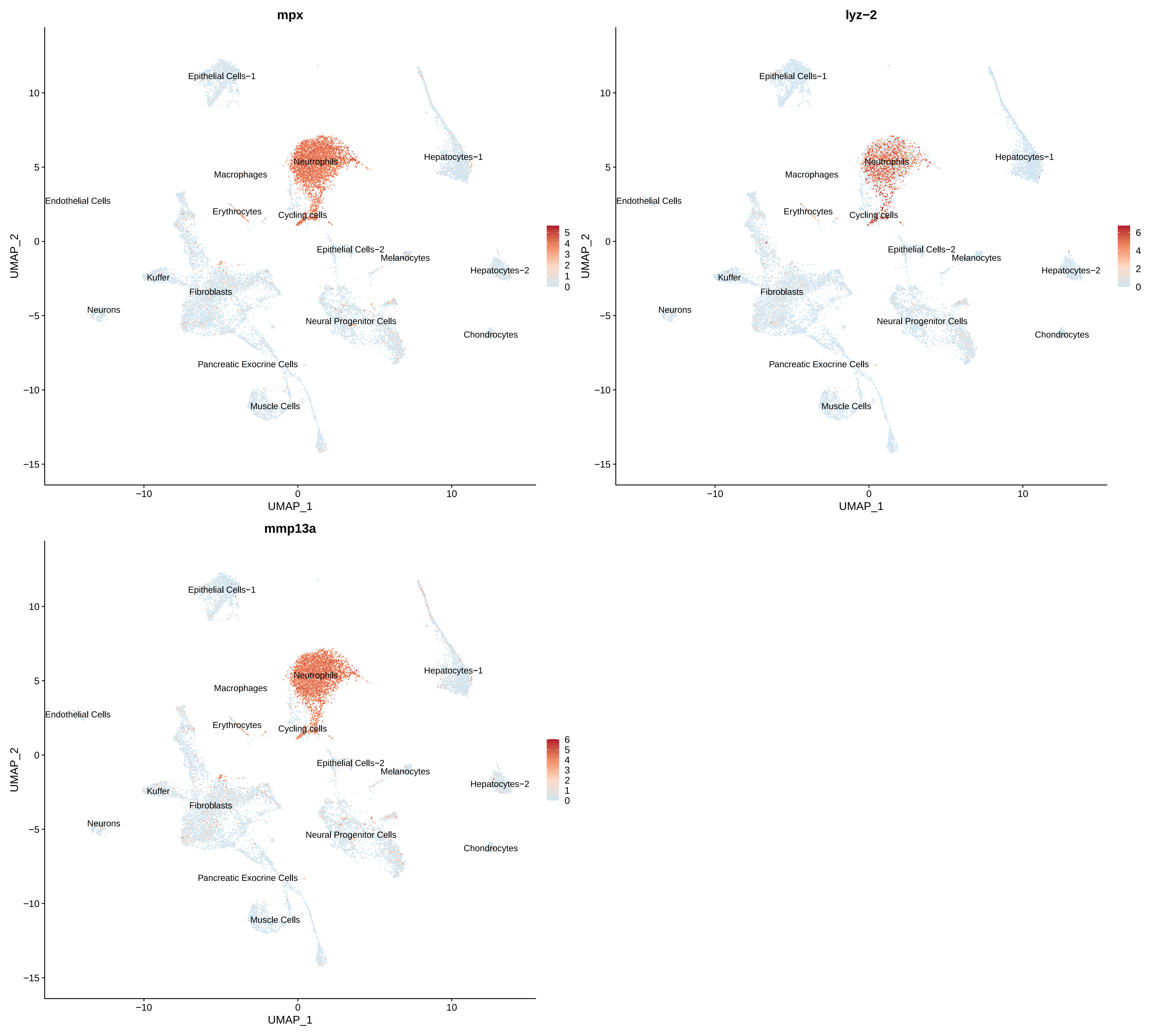
mpx
Epithelial Cells−1
Neutrophils
Hepatocytes−1
Macrophages
Endothelial Cells
Erythrocytes
Cycling cells
Epithelial Cells−2
Melanocytes
Hepatocytes−2
Kuffer
Fibroblasts
Neurons
Neural Progenitor Cells
Chondrocytes
Pancreatic Exocrine Cells
Muscle Cells
UMAP_1
UMAP_2
lyz−2
Epithelial Cells−1
Neutrophils
Hepatocytes−1
Macrophages
Endothelial Cells
Erythrocytes
Cycling cells
Epithelial Cells−2
Melanocytes
Hepatocytes−2
Kuffer
Fibroblasts
Neurons
Neural Progenitor Cells
Chondrocytes
Pancreatic Exocrine Cells
Muscle Cells
UMAP_1
UMAP_2
mmp13a
Epithelial Cells−1
Neutrophils
Hepatocytes−1
Macrophages
Endothelial Cells
Erythrocytes
Cycling cells
Epithelial Cells−2
Melanocytes
Hepatocytes−2
Kuffer
Fibroblasts
Neurons
Neural Progenitor Cells
Chondrocytes
Pancreatic Exocrine Cells
Muscle Cells
UMAP_1
UMAP_2
